# Unlocking the Door of Boosting Biodirected Structures for High‐Performance VN*_x_*O*_y_*/C by Controlling the Reproduction Mode

**DOI:** 10.1002/advs.201903276

**Published:** 2020-01-21

**Authors:** Ting Li, Jing Wang, Xia Li, Liang Si, Sen Zhang, Chao Deng

**Affiliations:** ^1^ Key Laboratory for Photonic and Electronic Bandgap Materials Ministry of Education College of Chemistry and Chemical Engineering Harbin Normal University Harbin 150025 Heilongjiang China; ^2^ Department of Biological Science and Engineering Modern Testing Center Harbin Normal University Harbin 150025 Heilongjiang China; ^3^ College of Materials Science and Chemical Engineering Harbin Engineering University Harbin 150001 Heilongjiang China

**Keywords:** aqueous Zn‐ion batteries, biodirected structure, reproduction mode, vanadate oxynitride

## Abstract

Diverse reproduction modes of bio‐organisms open new intriguing opportunities for biochemistry‐enabled materials. Herein, a new strategy is developed to explore biodirected structures for functional materials via controlling the reproduction mode. Yeast with sexual or asexual reproduction mode are employed in this work. They result in two different biodirected structures, from bowl‐like hollow hemisphere to “bubble‐in‐sphere” (BIS) structure, for the VN*_x_*O*_y_*/C composites. Benefitting from the hierarchical structure, nanoscale particles and conductive biomass–derived carbon base, both VN*_x_*O*_y_*/C biocomposites achieve high power/energy density, good reliability, and excellent long‐term cycling stability in aqueous Zn‐ion batteries. Deep investigations further reveal that different biodirected structures greatly influence the electrochemical properties of biocomposites. The bowl‐like structures with thin shells and folded double layers achieve larger surface area and more active sites, which ensure their faster kinetics and better high rate capability. The BIS structures with a more compact assembly and higher stack capability are favorable to the better energy storage. Therefore, this work not only introduces a new clue to boost biodirected structures for functional materials, but also propels the development of Zn‐ion batteries in diverse applications.

## Graphical abstract

Herein, a new strategy is developed to boost biodirected structures for functional materials via controlling the reproduction mode. Two different structures of bowl‐like hemisphere and “bubble‐in‐sphere” (BIS) are successfully constructed for VN*_x_*O*_y_*/C. Different features of diverse biodirected structures ensure the applications of high‐performance VN*_x_*O*_y_*/C in broad fields.

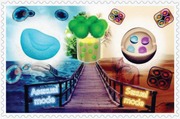

## 1 Introduction

As important energy storage systems, lithium‐ion batteries (LIBs) and sodium ion battery (SIBs) have attracted great attentions in the past decades. Much effort has been devoted to developing different materials and strategies for LIBs and SIBs. Many electrode materials have been extensively studied, such as the metal oxide compounds,^1^ polyanions,^2^ alloy materials,^3^ carbon‐based materials,^4^ etc. However, the increasing concerns on product price, battery safety, energy efficiencies, and so on greatly restrict their developments. Compared with these nonaqueous batteries, the high intrinsic safety and facile manufacturing of the aqueous rechargeable batteries, such as the aqueous lithium/sodium batteries (e.g., LiMn_2_O_4_/LiV_3_O_8_, Na_0.44_MnO_2_/NaTi_2_(PO_4_)_3_),^5^ metal‐ion (e.g., Mg^2+^, Zn^2+^, Ca^2+^, and Al^3+^) batteries,^6^ alkaline or acid batteries (e.g., Ni/MH and Cd/Ni, Pd‐acid),^7^ etc. have attracted increased attentions. Among these systems, the Zn‐ion batteries (ZIBs) have attracted particular interests due to their abundant resource, low cost, easy manufacturing, and high theoretical energy densities.

To date, the design and fabrication of high‐performance cathodes are one of the most important issues for Zn‐ion batteries. A variety of materials, including the manganese based oxides (e.g., α‐MnO_2_, Mn_2_O_3_, and Mn_3_O_4_),^8^ Prussian blue (PB) and its analogy (e.g., CuHCF, ZnHCF),^9^ polyanions (e.g., Na_3_V_2_(PO_4_)_3_, LiV_2_(PO_4_)_3_),^10^ vanadium based composites, etc. have been employed as cathode materials for ZIBs. Among these candidates, the vanadate based complex with large interlayer/tunnel spaces and multiple valence states show great superiority on ion transport and energy storage. Since the introduction of the V_2_O_5_/Zn system in 2016, a series of vanadium oxides, such as the V_2_O_5_,^11^ Zn_2_V_2_O_7_,^12^ H_2_V_3_O_8_,^13^ LiV_3_O_8_,^14^ Na_0.33_V_2_O_5_,^15^ VO_2_,^16^ V_2_O_5_ *n*H_2_O,^17^ etc., have been successfully applied as cathode materials in ZIBs. Very recently, a new vanadate based complex, i.e., the vanadate oxynitride (VN*_x_*O*_y_*), have been introduced as cathodes by Zhou's^18^ and Yang's^19^ groups. The abundant vacancies/defects induced by the substitution of low‐valent oxygen with high‐valent nitrogen provide more efficient ion diffusion channels and effectively improved their properties. However, the fast developments of the vanadate based composites are greatly restricted by their inherent low conductivity. Therefore, it is necessary to explore effective strategies to overcome these obstacles and propel their development.

Tailoring functional materials into hierarchical structures is an important strategy to maximum realizing their functional properties. Benefitting from the high diversity of the biological organisms and molecules, the biodirect synthesis shows great superiorities on exploring novel architectures. A large variety of functional materials with outstanding properties have been constructed based on diverse biological organisms, such as the virtus,^20^ DNA,^21^ cells,^22^ spores,^23^ plant,^24^ etc. For example, Zhou et al. constructed 3D A_3_V_2_(PO_4_)_3_/C (A = Li, Na) foams by assembly of elastin‐like polypeptides and obtained the ultrafast rate capabilities as applied in lithium/sodium ion batteries.^21^ Sun et al. designed a “self‐breathable” structure based on *Lycoperdon bovista* (LB) spore and built fast electron/ion transport highways inside the aurilave‐like structure.^25^ Zhang et al. in situ encapsulated the iron based complex into the egg yolk derived nanotubes to achieve superior capacitive performance.^26^ Our group has also engaged in building high‐performance electrodes on the basis of the biodirected strategies.^22^, ^27^, ^28^ For instance, we have constructed 3D hybrid foams and hollow spheres for the pyrophosphate composites based on fungus (*Auricularia*) and microalgae, respectively. Both biocomposites achieved fast kinetics and superior properties as applied in sodium ion batteries.^22^, ^28^ Moreover, a series of the architectures, from 3D hollow hexahedron to 2D hierarchical nanosheets, have also been constructed based on the fern spore to improve the performance of Na_7_V_3_(P_2_O_7_)_4_ composite.^27^

However, most present biodirected strategies are greatly restricted by the features of the bio‐organisms. Particularly, the biodirected structures closely depend on the intrinsic configuration of the bio‐organisms. To date, most of the reported biocomposites concentrate on relative simple structures, such as the hollow sphere, nanotube, nanosheet, foam, etc.^20^, ^21^, ^22^, ^23^, ^24^, ^25^, ^26^, ^27^, ^28^ It is still a virgin place to build intricate hierarchical structures via the biodirected route until now. Accordingly, it is necessary to carry out deep investigations on the bio‐organisms to explore new opportunities. It is well known that the configurations of the bio‐organisms change greatly at some special stages, such as the growth, the separation, the reproduction, etc. The various structures of the bio‐organisms in their whole lives provide a new clue to explore the novel biodirected structures. Particularly, the reproduction process, which is a very special period of the bio‐organisms, will result in novel structures for biodirected materials. Unfortunately, the relative studies are very rare up to now.

Following this viewpoints, for the first time, we carried out a new attempt to construct intricate hierarchical structure by controlling the reproduction mode. As a concept‐of‐proof study, we use the low‐cost and easily‐cultivated yeast, i.e., *Sacharomyces cerevisiae* (*S. cerevisiae*), as the biotemplates. Two different routes, i.e., the sexual or the asexual mode, are controlled to direct their different growth processes. As a result, two intricate hierarchical structures of the bowl‐like folded hollow hemisphere and the bubble‐in‐sphere (BIS) hierarchical structure are successfully constructed. In this study, the vanadate oxynitride (VN*_x_*O*_y_*) is employed as the electroactive materials and the VN*_x_*O*_y_*/C composites with diverse biostructures are constructed. The influences of different biostructures on the electrochemical performance of VN*_x_*O*_y_*/C composites are investigated. Moreover, the flexible Zn‐ion batteries were fabricated based on the prepared VN*_x_*O*_y_*/C biocomposites. Their superior energy or power densities and cycling stability ensure the applications in multiple fields.

## 2 Results and Discussion

### 2.1 Controlling the Reproduction Mode to Build Intricate Biodirected Structures

**Figure 1:**
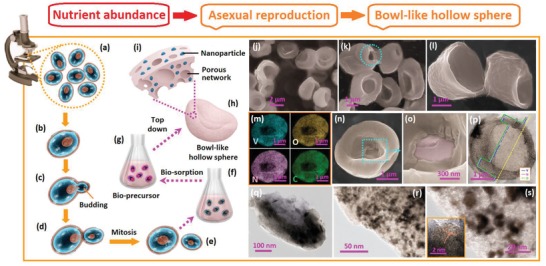
a–e) Schematic illustrations of asexual reproduction process of the yeast. f) Scheme of biosorption process and g) prepared bioprecursors. h) The illustrations of VN*_x_*O*_y_*/C with bowl‐like structure. i) The enlarged image emphasizes the porous shell with the enwrapped nanoparticles. j–l) SEM images of the bowl‐like particles. n) The image of a partial broken bowl‐like particle and o) enlarged image of its central broken parts. m) EDX element mapping results. p) TEM image of one bowl‐like hollow sphere with line‐scan element distribution results. q) TEM image of one piece of fragment. r,s) Enlarged images of the shells with highly porous networks and enwrapped nanoparticles. In (s), HRTEM image of one nanoparticle is displayed as insert. In (k), one broken part on the shell is emphasized by the blue dot circle.

**Figure 2:**
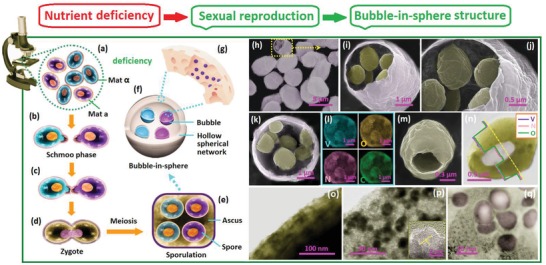
a–e) Schematic illustration of the sexual reproduction process of the yeast. f) Schematic illustrations of the bubble‐in‐sphere (BIS) structure. g) The enlarged image emphasizes the porous shell with enwrapped nanoparticles. h) Low‐resolution SEM image of the BIS structured particles. i,j) Enlarged images of one broken particle. k) Sectional image of one broken particle and l) EDX element mapping result. m) SEM image of one small bubble inside the sphere and n) TEM image with EDX line‐scan results. o) The partially enlarged TEM images of the bubble shell filled with pores. p,q) Enlarged images show the highly porous network and enwrapped nanoparticles. In (p), the HRTEM image of one nanoparticle is shown as insert.

The construction of the intricate structures proceeds along a biodirected process with controllable reproduction mode. As displayed in **Figures** **1** and **2**, the *S. cerevisiae* cells follows two different reproduction modes under different cultivated conditions.^29^ On the one hand, the asexual mode will take place in the presence of abundant nutrients (Figure 1a–e). In this case, the cells reproduce in the budding form. The small buds initially grow on the mother cell (Figure 1c), which is divided into a new one as it maturates with the mitosis (Figure 1d,e); On the other hand, the sexual mode will take place in some special conditions, such as starved for nitrogen, high pressure, nutrient deficiency, etc. In extreme conditions, such as the complete absence of nitrogen or the presence of a nonfermentable carbon source, the diploid cells will undergo the meiosis and sportulate (Figure 2a–d).^29^ In this case, the ascospores, which are composed of the clusters of four or eight spores within a single mother cell (i.e., the ascus), are constructed (Figure 2e). Through controlling the cultivated conditions, we successfully control the reproduction modes of the yeast. Detailed information is supplied in Section S‐1‐1 in the Supporting Information). The cultivated yeast cells are employed as the biotemplates to build the bioprecursors (Figure 1f,g, details in Section S‐1‐2 in the Supporting Information). The good bioactivity and super self‐assembling capability of the yeast cells enable their highly efficient solution adsorption ability and good ion capture capability. After the high‐temperature treatments, the VN*_x_*O*_y_*/C composites with different biodirected structures are prepared (Details in Section S‐1‐3 in the Supporting Information).

The morphologies of the biocomposites are investigated. First, the bowl‐like folded hollow hemispheres are observed as the yeast cells reproduce in the asexual mode. As observed in Figure 1j–l, the particles have folded hollow hemispherical structure and the uniform size of 2–3 µm. Enlarged images of a broken particle indicate its central hollow nature (Figure 1k) and the thin shell with the thickness of 50–150 nm (Figure 1n,o). Transmission electron microscope (TEM) observations certify the highly porous nature of the shell (Figure 1q–s). Accordingly, both factors, i.e., the big central hollow nature and the thin and porous shell, enable the folding of the hollow spheres. The formation of this architecture is associated with the natural feature of the yeast. As a typical eukaryotic cell, the yeast cell is composed of the tough shell and the vulnerable core. When the high‐temperature treatment is employed, the core of the cell is subjected to destruction and the shell of the cell tends to carbonize into the porous network. The thin and porous shells of the big hollow sphere tend to fold into the double layered structure and construct the “bowl‐like” structure. The uniform distributions of V, N, O, and C elements in the EDS results demonstrate the homogenous distributions of vanadate oxynitride inside the biocomposite (Figure 1m,p). More clear characterizations are achieved by TEM observations. As displayed in Figure 1r,s, a large amount of small nanocrystals (5–10 nm) are enwrapped inside the matrix of the porous network. HRTEM results further confirm the well‐defined lattice fringes with lattice spacings of 0.22 nm (inserts of Figure 1s), which corresponds well to (111) planes of the vanadate oxynitride.^18^, ^19^

Compared with the bowl‐like structure that follows the asexual reproduction, a more complex architecture is obtained when the sexual reproduction mode is followed. As observed in Figure 2h–k, the large central hollow spherical framework with the size of 4–5 µm and the shell thickness of 200–300 nm are achieved for the biocomposites. Enlarged scanning electron microscope (SEM) observations (Figure 2i,j) demonstrate the presentence of small spheres with the size of 1–2 µm inside the framework. In addition, SEM and TEM observations of a single small sphere suggest its central hollow nature with the shell thickness of 200–300 nm (Figure 2m,n). Accordingly, the above results demonstrate that the “bubble‐in‐sphere” hierarchical structure has been constructed, which well copies the ascospores of the feast. As illustrated in Figure 2a–e, the ascospores are the special living state for the yeast, which usually appear in the sexual reproduction. They come from the meiosis of the zygote and are composed of the ascus and the small spores (Figure 2e). To the best of our knowledge, it is the first time that the ascospores are applied in the biodirected synthesis. In the ascospores, the shell of the ascus is relative thick and tough; and the immature spore has smaller size and thicker shell than those from the asexual reproduction. The cross sectional image of a broken particle and the corresponding EDS mapping results demonstrate the uniform distributions of the V, N, O, and C elements across both the outside spherical framework and the inside small bubbles (Figure 2k,l). EDS line‐scan results further clarify the uniform element distributions on the internal small hollow bubble (Figure 2n). Enlarged TEM observations further demonstrate the porous structures of its shell (Figure 2o). Compared with above bowl‐like sample, the thicker shell with inferior porosity is detected for the BIS sample. It is associated with the strong structure of the natural ascropores. In addition, enlarged TEM images certify the nanoparticles enwrapped in the carbon network (Figure 2p,q). The well‐resolved lattice fringe on HRTEM image (inset of Figure 2p) identifies the existence of vanadate oxynitride nanoparticles.

**Figure 3:**
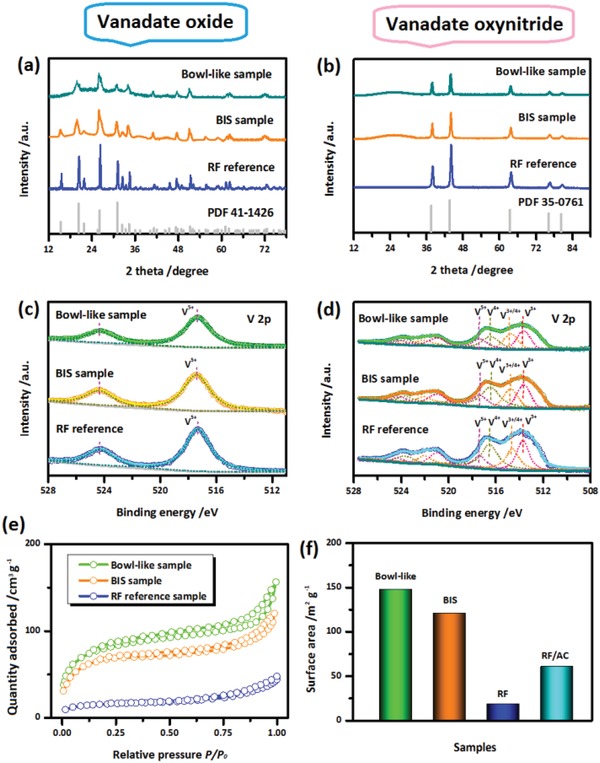
a,b) XRD patterns and c,d) XPS spectroscopy of the a,c) V_2_O_5_ and b,d) VN*_x_*O*_y_* based samples. e) Nitrogen adsorption/desorption curves and the corresponding surface areas of the VN*_x_*O*_y_* based samples. f) The RF/AC sample is prepared by mixing the RF sample with the activated carbon to achieve the same carbon content as the biocomposites.

More evidence about the phase and composition of the biocomposites with different biodirected structures are provided by X‐ray diffraction (XRD), X‐ray photoelectron spectroscopy (XPS), and element analysis results. In this study, the vanadate oxynitride (VN*_x_*O*_y_*) are used as electroactive materials to build biocomposites. For comparison, the intermediate product during calcination, i.e., the vanadate oxide (V_2_O_5_) based samples with the same kind of biodirected structures, are also investigated (Section S‐1‐3, Supporting Information). XRD patterns of the both VN*_x_*O*_y_* and V_2_O_5_ composites with different biodirected structures are investigated. **Figure** **3**a,b shows the XRD patterns of both VN*_x_*O*_y_* and V_2_O_5_ based samples. In both cases, the reference samples (RF) were also employed for comparison. The RF reference samples are prepared by treating inorganic component in the same procedure (Section S‐1‐4, Supporting Information). All the reflections of the samples can be well indexed to their standard patterns and are in good coincidence with previous studies.^11^, ^18^, ^19^ Compared with the sharp peaks of the RF reference samples, the biocomposites exhibit the broader and lower peaks, which are associated with their smaller particles with inferior crystallinity. For all the biocomposites, the broad peaks in the range of 20–40° are detected, which can be attributed to the highly porous carbon matrix inside the biocomposites.

The XPS analysis was carried out to investigate the element state of vanadate in different samples. The similar spectra were achieved for samples with the same kind of the vanadate based composite. As displayed in Figure 3c, the V_2_O_5_ based samples exhibit two peaks at 517.3 and 524.4 eV, which can be well identified as V 2p_3/2_ and V 2p_1/2_ of V^5+^.^30^ While, a series of peaks positioned at ≈513.4, 514.6, 516.5, and 517.3 eV in V2p_3/2_ spectra are detected for VN*_x_*O*_y_* based samples (Figure 3d). They correspond to the V^3+^, V^3+/4+^, V^4+^, and V^5+^, which demonstrates the mixed state of V element in the vanadate oxynitride composite. Similar phenomenon has been obtained in previous studies on vanadate oxynitride.^18^, ^19^ It has been attributed to the unavoidable surface oxidations. Based on the XPS and element analysis results, the approximate atomic ratios of vanadium, nitride and oxide for the vanadate oxynitrides are achieved. The bowl‐like and BIS structured VN*_x_*O*_y_* composites achieve the approximate atomic ratios (V:N:O) of 1.0:0.86:0.21 and 1.00:0.88:0.18, respectively. In fact, VN*_x_*O*_y_* is very susceptible to surface oxide in the ambient air, which leads to the inexistence of the completely stoichiometric vanadium nitride in ambient air. Thus, it is often used the “vanadate oxynitride” to describe this kind of materials in the related previous studies.^18^, ^19^

The nitrogen adsorption/desorption measurements were employed to provide a clearer comparison on the surface characteristics between the samples. As displayed in Figure 3e,f, VN*_x_*O*_y_* based bowl‐like sample achieves the highest surface area among the samples, which is followed by the BIS structured one. In contrast, the RF reference sample achieves much lower surface area values than the biodirected composites. Similar trend is also detected for the V_2_O_5_ based samples (Figure S1, Supporting Information). The poor surface characteristics of the RF reference samples are associated with the absence of carbon (Figure S2, Supporting Information) and the large particle in block nature (Figure S3, Supporting Information); While, the nanoscale particles and highly porous carbon network of the biocomposites result in their higher surface area. In addition, the RF/AC reference samples are prepared by mixing the RF reference sample with the activated carbon to achieve the same carbon content as the biocomposites (Section S‐1‐4‐ii, Supporting Information). Although the improved surface areas are obtained for RF/AC sample as compared with the pristine RF sample, the surface area of the RF/AC sample is still much lower than those of the biocomposites (Figure 3f). On the other hand, with similar carbon contents, the different surface characteristics between the biocomposites are associated with their different structures. As discussed above, the porous, uniform and thin shells of the bowl‐like structured sample enable it to achieve higher surface characteristics; while, the thicker shell with inferior porous structure of the BIS samples lead to the lower surface area. Accordingly, the above results demonstrate both kinds of biodirected structures can significantly promote the surface areas for the functional materials. Particularly, the thin and highly porous shell enables the bowl‐like sample to achieve high porosity and ensure its fast kinetics; while, the presence of internal bubbles enable the BIS sample to achieve a compacter structure. After dissolving the active materials from the biocomposites, the higher tap density is achieved for the BIS structured carbon base than the bowl‐like one (Figure S4, Supporting Information). It indicates the better stack capability of the BIS biodirected structure, which ensures its better energy storage capability.

Based on above results, two kinds of the biodirected structures, i.e., the bowl‐like and the BIS structures have been successfully constructed by controlling the reproduction mode. Both kinds of the biodirected composites are composed of the porous carbon network and in situ formed active material nanoparticles, which ensure their common advantages on fast electron/ion transport and superior electrochemical properties. Deep investigations reveal that features of different biodirected structures greatly influence their properties. On the one hand, the bowl‐like structure with the thin shell and highly porous structure facilitate the fast kinetics and superior high rate capability, which is favorable to the applications toward high power density; on the other hand, the BIS structure with the intricate hierarchical and compact structure facilitates the high stacking and energy storage, which is favorable to the applications toward high energy density. Therefore, the biodirected strategy with controllable reproduction mode provides a new clue to design and fabricate diverse hierarchical structures.

### 2.2 Ion Transport Kinetics and Electrochemical Performance

**Figure 4:**
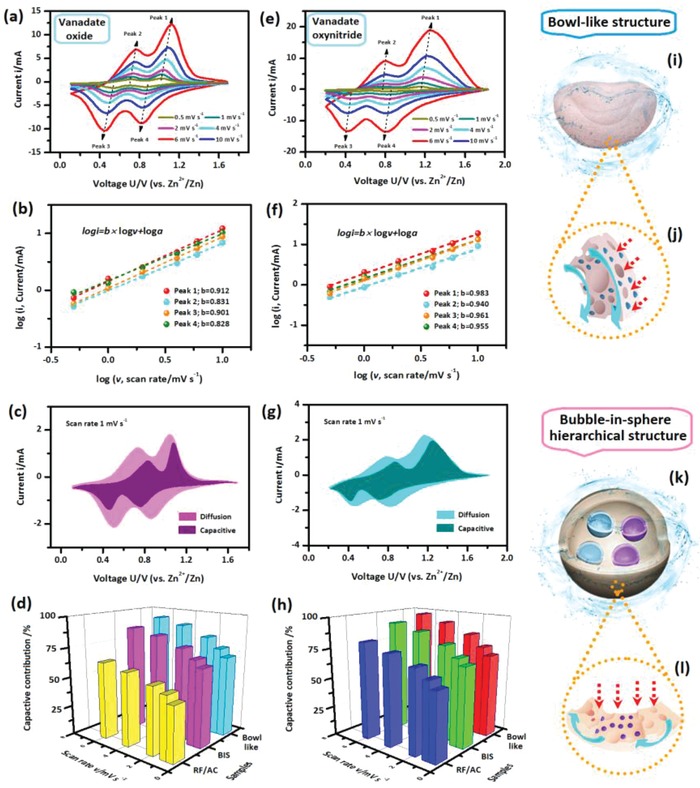
a,e) CV curves and b,f) the corresponding plots of the log *i* versus log *v* at the peak 1 to 4 of the a,b) V_2_O_5_ and e,f) VN*_x_*O*_y_* based samples. c,g) CV profiles of the c) V_2_O_5_ and e) VN*_x_*O*_y_* BIS samples with the capacitive contribution ratios denoted by the shaded region. d,h) The calculated capacitive ratios of the d) V_2_O_5_ and h) VN*_x_*O*_y_* based bowl‐like and BIS biocomposites and the RF/AC reference sample at different scan rates. i–l) Schematic illustration of the i,j) bowl‐like and k,l) BIS structure with the i,k) easy electrolyte penetration and j,l) fast electronic/ion pathways. In (j) and (l), the blue arrow represents the electron transport pathways and the red arrow represents the ion transport pathway. In (i) and (k), the blue transport sphere surrounding the biodirected particles illustrates the easy electrolyte penetration inside the particles.

To evaluate the advantages of the biodirected samples, their electrochemical behaviors were investigated. First, we analyze the capacitance contributions of the samples. **Figure** **4**a,e illustrates the cyclic voltammetry (CV) curves of the V_2_O_5_ (a) and VN*_x_*O*_y_* (e) based BIS structured samples at various scan rates. For both kinds of the biocomposites, two pairs of the redox peaks are detected in the CV curves, which are in good agreement with the previous reports.^11^, ^18^, ^19^ As the scan rate is increased, the shapes of the CV curves is maintained and the redox peaks shift to lower or higher potentials regularly. Generally, the currents in the CV curves are believed to be originated from two independent parts: the surface‐induced capacitive process and the diffusion‐controlled process. For both kinds of the biocomposites, the *b* values from the linear fitting of log *i* versus log *v* are in the range of 0.5–1 (Figure 4b,f). It indicates the synergistically controlling from both capacitive and diffusion behaviors. Based on the current (*i*) and the scan rate (*v*), the capacitive contribution (*k*_1_v) and the ion diffusion contribution (*k*_2_v^1/2^) can be calculated (Details in Section S‐6 in the Supporting Information). As emphasized by the shaded regions in CV curves (Figure 4c,g), the capacitive contributions are calculated. Figure 4d,h summarizes the capacitive contributions of V_2_O_5_ (d) and VN*_x_*O*_y_* (h) based bowl‐like and BIS biocomposites and the corresponding RF/AC reference samples at all various scan rates. When the scan rate is increased, the capacitive contributions of all the samples are increased correspondingly, suggesting the enhanced dominant role of the capacitive controlled contributions. For each kind of biocomposites, the bowl‐like structured sample obtains the highest capacitive contributions at all the scan rates, which is followed by the BIS structured sample. In contrast, the RF/AC reference sample obtains the lowest values among the samples. In addition, the increased differences between the samples are detected as the scan rate is increased. The results demonstrate the faster ion transport and superior kinetics of both biocomposites as compared with the reference one. On the other hand, when the same structure is employed, the VN*_x_*O*_y_* based samples achieve the larger capacitive contributions than the corresponding V_2_O_5_ based ones at the same scan rate. It certifies the faster kinetics and better high rate properties of the VN*_x_*O*_y_* based samples as compared with the V_2_O_5_ based ones. According to previous report, it is associated with the rapid surface redox reaction on the surface of the vanadate oxynitrides.^18^, ^19^ Combined above results, it demonstrates that the biodirected samples have superiority on the capacitive characters than the RF/AC reference samples. It facilitates the fast ion transport and good charge/discharge properties, especially at high current densities. As illustrated in Figure 4i–l, the fast kinetics of the biodirected samples is associated with their unique structure. According to above results, both kinds of the biocomposites are composed of the highly porous and conductive network with embedded nanoscale particles. The hierarchical structure facilitates easy electrolyte penetration and is favorable to the fast electron/ion transport capability. Particularly, the central hollow and thin shell of the bowl‐like structure (Figure 4i) enables it to achieve the highest capacitive contributions at all current densities, which ensure its superior high‐rate performance.

**Figure 5:**
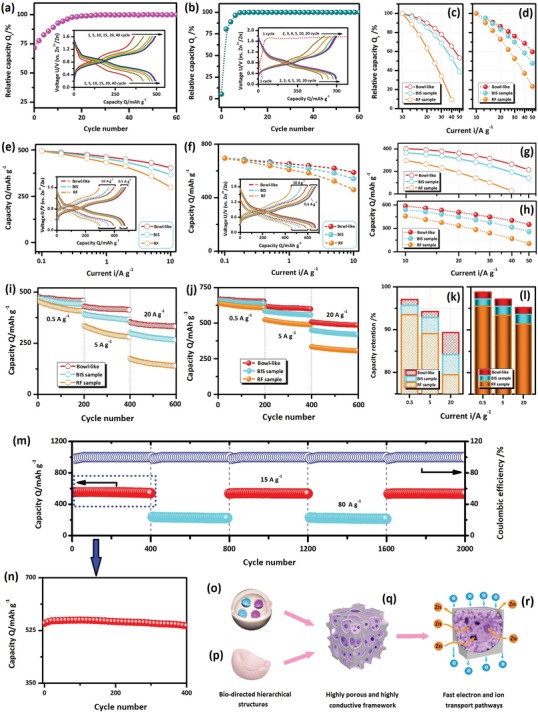
a,b) Initial charge/discharge properties and e,f) rate capability of a,e) V_2_O_5_ and b,f) VN*_x_*O*_y_* based bowl‐like samples at relative low current densities (0.1–10 A g^−1^). g,h) Comparisons of discharge capacities and c,d) relative rate capacities of c,g) V_2_O_5_ and d,h) VN*_x_*O*_y_* based samples at relative high current densities (10–50 10 A g^−1^). i,j) Cycling properties and k,l) capacity retentions of i,k) V_2_O_5_ and j,l) VN*_x_*O*_y_* samples at current densities of 0.5, 5, and 20 A g^−1^. m) Long‐term high‐rate properties of VN*_x_*O*_y_* bowl‐like samples at alternative 15 and 80 A g^−1^. n) Cycling properties of the sample at 15 A g^−1^ during the initial four hundred cycles. o–r) Schematic illustrations of o,p) biodirected structures with q,r) fast electron/ion pathways. In (a) and (b), the charge/discharge curves are displayed as inserts; in (e) and (f), charge/discharge curves at typical current densities are showed as inserts.

Inspired by the fast kinetics and high capacitive contributions, the electrochemical properties of the samples are investigated. To ensure the reliability of the results, all the electrochemical tests were carried out on the samples with three times to ensure the good repeatability of the results. First, the galvanostatic charge/discharge properties of the samples in the initial sixty cycles are investigated. To clarify the difference between different vanadate composites, the relative capacity (*Q*_c_), which is the ratio of the capacity in a certain cycle to that in the sixty cycle, are calculated (Section S‐5‐ii, Supporting Information). As displayed in **Figure** **5**a, continuously capacity increase is observed for V_2_O_5_ bowl‐like sample in the initial twenty cycles, indicating the gradual increased utilization process of active materials. In contrast, the dramatic capacity increase is observed for the VN*_x_*O*_y_* bowl‐like sample from the first to the second cycle, which follows the quick capacity increase within the initial five cycles (Figure 5b). This special phenomenon on the first cycle of VN*_x_*O*_y_* has also been detected in previous studies by Yang's group.^19^ They have carefully investigated the mechanism for this phenomenon by a series of techniques including the high‐angle annular dark‐field (HAADF) atomic‐resolution observation, ultrathin sectioning TEM, operando XRD analysis, X‐ray absorption near‐edge structure (XANES), etc. Based on the results, they demonstrated the appearance of a new phase with large defects during the initial charge, which was believed to be the crucial factor for the superior performance of VN*_x_*O*_y_*. In present study, we investigate the change of the VN*_x_*O*_y_* composite in the first cycle and obtain the similar results as previous studies. First, the change of the composition for the VN*_x_*O*_y_* after the initial fully charging is investigated. Based on the XPS and element analysis results, the approximate atomic ratios of V, N, and O for the fully charged product of BIS sample is about 1.00:0.19:2.12. Compared with the pristine composition of the BIS sample, which has the V:N:O ratio of 1.00:0.88:0.18, the significant increased oxygen and decreased nitride contents are detected. The result indicates the pristine VN*_x_*(O_poor_)*_y_* is convert to the VN*_x_*(O_rich_)*_y_* after the initial charging. Next, the HRTEM observation result indicates the absence of the well‐resolved lattice fringe for fully charged product (Figure S5, Supporting Information). The disordered feature indicates the existence of defects in the fully charged product. Accordingly, both above results indicate the formation of the new product (VN*_x_*(O_rich_)*_y_*) with large defects after the initial charging, which is schematically illustrated in Figure S6b in the Supporting Information. The result is in good coincided with the previous study.^19^ In the following cycles, the Zn ion intercalations take place on this new defect‐rich product. This defective‐rich new product enables the high reversible Zn ion transport and promotes the fast electrochemical activation. Therefore, as compared in Figure 5a–h, the VN*_x_*O*_y_* based sample achieves the superior electrochemical performance and higher activity than the V_2_O_5_ based one.

Based on above discussions, the very low columbic efficiency of the VN*_x_*O*_y_* in the initial cycle can also be well explained. As detected in Figure 5b, the VN*_x_*O*_y_* sample exhibit the slope initial discharge curve without obvious plateaus and very low capacity (35.8 mAh g^−1^). The poor performance is associated with the structure of the pristine VN*_x_*O*_y_*. As illustrated in Figure S6a in the Supporting Information, the pristine VN*_x_*O*_y_* has close‐packed face‐centered cubic structure, which greatly limits the Zn ion diffusion and leads to the poor electrochemical performance. Based on above discussions, in the initial charging process, the high‐valent anion nitrogen (N^3−^) was partially substituted by the low‐valent anion oxygen (O^2−^). It results in the new product with anion‐disordered structure and abundant defects, as illustrated in Figure S6b in the Supporting Information. The conversion reaction during the initial charge gives rise to the abnormal features of the initial charge curve, which exhibits long quasiplateau at high potentials. Accordingly, both the poor discharge performance for the pristine VN*_x_*O*_y_* and the conversion reaction in the initial charge process lead to the very low coulombic efficiency of VN*_x_*O*_y_* sample during the first charge/discharge cycle.

The rate capabilities of all the samples are investigated. The properties of the samples at relative low current densities are investigated first. Figure 5e,f displays the changes of the capacities for the V_2_O_5_ (e) and VN*_x_*O*_y_* (f) based samples at the current densities from 0.1 to 10 A g^−1^. As the current density increases, the capacities of all the samples decrease correspondingly. For both vanadate composites, the bowl‐like samples achieve the highest capacities at all the current densities, which are followed by the BIS structured ones; while, the RF reference samples obtain the lowest capacities and the fastest capacity deteriorations among the samples. The charge and discharge curves of all the samples at two typical current densities were employed to show their different behaviors (insets of Figure 5e,f). At both current densities, the bowl‐like sample of both vanadate composites exhibits the highest discharge potentials and the lowest difference between the charge/discharge potentials, indicating their lowest polarization and best reversibility; In contrast, the lowest capacities and the highest polarizations of the RF/AC reference samples indicate their poor electrochemical reversibility among the samples. Accordingly, the above results demonstrate the superior high rate capabilities of the biodirected samples. Next, the properties of the samples at higher current densities are investigated. Figure 5g,h displays the discharge capacities of the samples at the current densities of 10–50 A g^−1^. To clarify the difference between the samples, the relative rate capacities (*Q*_r_) of all the samples were calculated based on the ratio of the capacity at a certain rate to that at 10 A g^−1^ (Section S‐5‐iii in the Supporting Information, Figure 5c,d). As displayed in Figure 5c,d,g,h, the differences between different structured samples are enlarged as the current density increases. When the same structure is employed, the VN*_x_*O*_y_* based samples achieve the higher discharge capacities and relative rate capacities than the corresponding V_2_O_5_ based ones. In addition, the differences between the samples become more significant as the current density is increased. Therefore, the above results demonstrate the superior high rate capabilities of the VN*_x_*O*_y_* based samples than the V_2_O_5_ based ones.

The cycling properties of all the samples are investigated. Figure 5i,j displays the cycling performance of all the samples at three typical current densities of 0.5, 5, and 20 A g^−1^ in the low, mediate and high ranges. The capacity retentions (*C*_r_) of all the samples during the cycling are calculated (Section S‐5‐iv in the Supporting Information, Figure 5k,l). For each kind of the vanadate composite, the bowl‐like structured sample achieves the highest capacity retention and the best cycling stability among the samples, which are followed by the BIS structured samples; while, the RF reference sample obtains the lowest capacity retentions at all current densities, indicating its poorest cycling stability. On the other hand, when the same structured is employed, the VN*_x_*O*_y_* based samples achieve higher capacity retentions than the corresponding V_2_O_5_ samples at all the current densities.

Furthermore, the high‐rate long‐term properties of the VN*_x_*O*_y_* biodirected samples are studied. The VN*_x_*O*_y_* bowl‐like samples achieve the high capacity retentions of 97.4% (Figure 5m) with the high coulombic efficiencies near 100% during two thousand cycles at alternative current densities of 80 and 15 A g^−1^. The VN*_x_*O*_y_* sample after long‐term cycling is investigated. As displayed in Figure S7a in the Supporting Information, SEM observations demonstrate the well‐retained architectures for the VN*_x_*O*_y_* BIS sample after long‐term cycling. In addition, no obvious change is detected as comparing the XRD patterns of the samples before and after cycles (Figure S7b, Supporting Information). Therefore, the above results demonstrate the good structural and morphological stability of the VN*_x_*O*_y_* based sample during long‐term cycling, which contribute to their superior cycling stability.

More careful investigation reveals that the change of the capacity during high‐rate long‐term cycling is not uniform. As displayed in Figure 5n, the capacity increases slowly during the initial ninety cycles, and then turns to decrease as the cycle number further increases. The similar phenomenon has also been detected in other kinds of the electrodes during high‐rate cycling in many previous studies.^31^ Some possible factors, for example, the improved electrolyte penetration in the micropores of electrode by electrostatic force, the improved interface state of the electrode during change/discharge, etc. have been proposed to explain this phenomenon.^32^ However, the exact reason is still lack and further analysis for an indepth understanding is still carried out until now. All the previous studies demonstrate the existence of initial self‐adjustment process, which enables the electrodes to accommodate the fast charge/discharge process more perfectly.^31^ When the current density is large, this process is obvious. When the current density is low or the electrodes have experienced the low‐rate cycling, such as the cases in Figure 5i,j, its influence turns to be inconspicuous. In our work, the capacity retentions of the high‐rate and long‐term cycling tests (Figure 5m) are calculated based on the ratio of the capacity obtained in the final cycle to the first cycle (Section S‐5‐iv, Supporting Information). The lower capacity of the first cycle leads to the higher capacity retentions of 97.4% at alternative high rate of 15 and 80 A g^−1^. If the maximum capacity, which is obtained at about 90th cycle is used, the lower capacity retention of 95.1% will be obtained. Although the existence of the small difference between two results, the high durability and good stability still certify the superior high‐rate long‐term cycling properties of the VN*_x_*O*_y_* based samples.

Combined above results, the VN*_x_*O*_y_* biodirected samples exhibit excellent electrochemical reversibility, superior high rate capability and stable long‐term cycling properties. Based on above discussions, these superior performance are associated with three aspects on their unique architecture and intrinsic structure (Figure 5o–r): First, the biomass derived carbon substrate have highly conductive and highly porous structures, which enable easy electrolyte penetration and fast ion and electron transport (Figure 5q); Second, the nanoscale particles of the active materials efficiently shorten the ion transport pathways and is favorable to the fast kinetics (Figure 5r); Third, the abundant vacancies/defects of the VN*_x_*O*_y_* enhance the electrochemical reversibility and facilitate the fast redox reaction.

### 2.3 Different Features of Diverse Biodirected Structures on Energy Storage or High‐Power Capability

**Figure 6:**
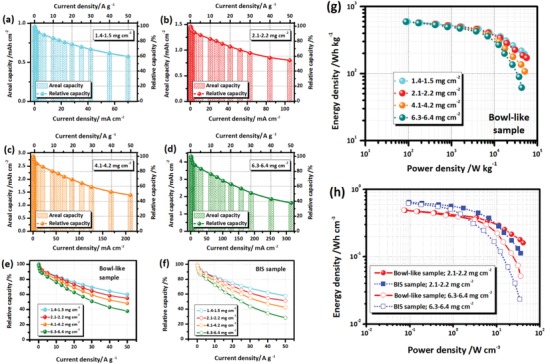
a–d) Rate capabilities of the VN*_x_*O*_y_* bowl‐like samples with different mass loadings. e,f) Comparisons of the relative capacities of the e) bowl‐like and f) BIS samples with different mass loadings at various current densities. g) Ragone plot of the bowl‐like sample with different mass loadings. h) Comparisons of the volumetric energy/power densities for the bowl‐like and BIS samples with low (2.1–2.2 mg cm^−2^) and high (6.3–6.4 mg cm^−2^) mass loadings.

The energy density and power density are crucial criterions for the secondary batteries toward practical applications. To clarify different features of diverse biodirected structures on energy storage or high‐power capability, the areal capacities and energy/power densities of the VN*_x_*O*_y_* based samples are investigated. First, the properties of the electrode with different mass loadings are investigated. The mass loading of the electrodes in above discussions are kept at 2.1–2.2 mg cm^−2^. To clarify the influence of the mass loading on the electrochemical properties, three other mass loadings, which are lower (1.4–1.5 mg cm^−2^) or higher (4.1–4.2 and 6.3–6.4 mg cm^−2^) than the previously used one, are employed for electrodes. The rate capabilities of the VN*_x_*O*_y_* based bowl‐like samples with different mass loadings are studied (**Figure** **6**a–d). When the current density is increased, the capacity of each sample decreases correspondingly. As the mass loading is increased, more significant deteriorations are detected for the samples with the increased current density. For example, the areal capacity of 1.46 mAh cm^−2^ was achieved for the bowl‐like structured VN*_x_*O*_y_* sample at the current density of 0.21 mA cm^−2^ (0.1 A g^−1^) with the mass loading of 2.1–2.2 mg cm^−2^. As the current density increases to 42 mA cm^−2^ (20 A g^−1^), the capacity of 1.07 mAh cm^−2^ was achieved, corresponding to 73.2% of the capacity at 0.21 mA cm^−2^. When the mass loading increases to 6.3–6.4 mg cm^−2^, the capacity of 4.32 mAh cm^−2^ was achieved at 0.63 mA cm^−2^ (0.1 A g^−1^), and 62.7% of the capacity is obtained as the current density increases to 126 mA cm^−2^ (20 A g^−1^). To evaluate the capacities at different current densities, the normalized areal capacity (*Q*_n_) is calculated based on the ratio of the areal capacity at a certain current density to that at the lowest current density (Section S‐5‐v, Supporting Information). The normalized areal capacities of the VN*_x_*O*_y_* based samples with different biodirected structures are summarized in Figure 6e,f. Both biodirected samples exhibit the deteriorations on the relative capacities as the current density increases. The faster deteriorations with increasing current density are detected for each kind of sample with the higher mass loading. When the same mass loading is employed, the BIS sample exhibits the faster capacity deteriorations than the bowl‐like sample as increasing the current density.

Next, the Ragone plot of the bowl‐like samples is studied to investigate the effects of the mass loading on the energy/power densities (Figure 6g). When the power density is lower than 3 kW kg^−1^, all the samples with different mass loadings exhibit similar behaviors. As the power density is increased, the energy densities of the samples decrease correspondingly with the increased differences between the samples. As the power density is higher than 20 kW kg^−1^, the samples with different mass loadings exhibit different features. For the samples with the higher mass loadings of 4.1–4.2 and 6.3–6.4 mg cm^−2^, dramatic energy density deteriorations are detected as the power density increases; while, the well retained energy density and slow deterioration are detected for the samples with low mass loadings (1.4–1.5 and 2.1–2.2 mg cm^−2^). The results demonstrate the low mass loading is favorable to the fast charge/discharge and high‐power capability of the electrodes.

On the other hand, the volumetric energy/power densities of the biodirected samples are investigated to clarify their different features on volumetric energy storage capability (Figure 6h). As the power density increases, the energy density deteriorations are detected for all the samples with both high (6.3–6.4 mg cm^−2^) and low (2.1–2.2 mg cm^−2^) mass loadings. For all the biodirected samples, the high mass loading samples exhibit faster energy density deteriorations than the low mass loading ones. When the same mass loading is employed, different behaviors are detected between the samples. When the power density is lower than 1 W cm^−3^, the BIS sample with both high and low mass loadings exhibit higher energy densities than the bowl‐like one with the same mass loading. However, the BIS samples exhibit faster deteriorations than the bowl‐like ones as the power density increases. When the power density is higher than 10 W cm^−3^, the energy densities of the BIS samples are much lower than the bowl‐like ones. Especially, the BIS sample with high mass loading achieves the lowest energy densities among the samples in the high power density range.

Combined above results, it demonstrates the high energy storage capability of the BIS sample in the low power density and the superior high rate capability of the bowl‐like sample in the high power density range. As discussed above, the different features of both biocomposites are associated with their different structures. The BIS sample composed of the hollow framework and the internal bubbles has compacter structure that facilitates to improve the volumetric energy storage capability. It results in its higher energy density under the low current densities. In contrast, the bowl‐like structure with thin shell and internal voids facilitates the fast kinetics and results in superior high power density. Therefore, the diverse features of different biodirected structures enable their superiorities in different field of applications.

### 2.4 Flexible Solid‐State Zn‐Ion Battery in Multiple Applications

**Figure 7:**
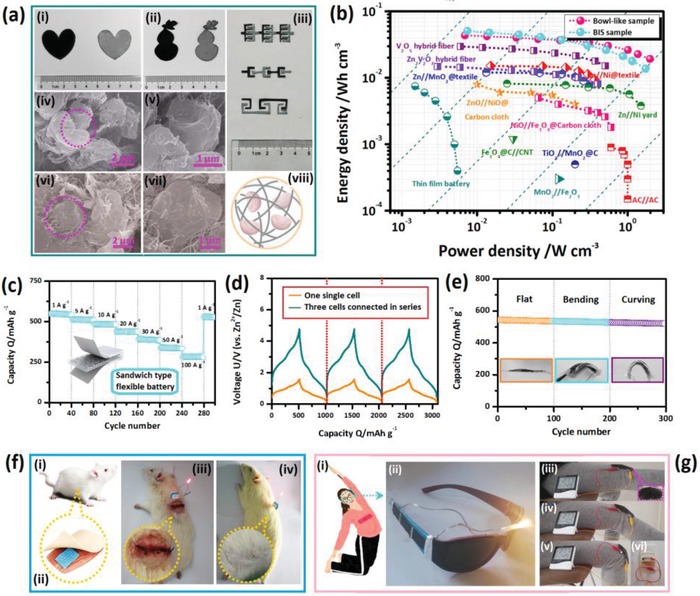
a) Digital photos and microstructure of the VN*_x_*O*_y_* /SWNT flexible electrodes. i–iii) The photos of the flexible electrodes tailored into various shapes; iv–vii) SEM images of the iv,v) bowl‐like and vi,vii) BIS structured flexible electrodes. vii) The schematic illustration of the structure of flexible electrode. b) Ragone plot of the sandwich batteries based on VN*_x_*O*_y_*/SWNT flexible electrodes. c) Cycling properties of the sandwich batteries. d) Galvanostatic charge/discharge curves of the microbattery. e) The cycling properties of the microbatteries during different outside deformations. f,g) The illustrations of the flexible batteries for practical applications of wearable g) electronic and f) implantable electronics. fI,ii) The schematic illustrations of the microbattery implanted into the SD rate. iii) SD rat with the lighted LED powered by the implanted microbattery after just implanting treatment and iv) after wounded is healed. gi) The schematic illustration of the sandwich battery for wearable electronics. gii) The LED light in smart glass model power by the sandwich type cells. giii–v) The electronic clock powered by the sandwich flexible battery attached to the trousers. gvi) the back of the electronic clock.

The superior properties of the biodirected VN*_x_*O*_y_* samples ensure their applications in broad fields. To investigate their potentials for practical applications, the flexible Zn‐ion batteries were fabricated based on the flexible biodirected composite/SWNT (carbon nanotube) cathodes, Zn nanosheet/SWNT anode (Figure S8, Supporting Information) and polymer electrolyte (Sections S‐1‐5 and S‐4‐1, Supporting Information). As displayed in **Figure** **7**aiv–vii, both kinds of biocomposite particles are encapsulated by the carbon nanotubes. The long and interconnected carbon nanotubes not only provide a highly conductive network that facilitates fast electronic transport (Figure 7aviii), but also ensure the construction of self‐supported flexible electrodes. The high pliability and good flexibility of the film electrodes enable it to be tailored into diverse shapes from simple structure such as the heart (Figure 7ai) and the gourd (Figure 7aii), to the complex and microtype shapes such as various interdigitation circuits (Figure 7aiii).

First, the electrochemical performances of the sandwich‐type flexible Zn‐ion batteries (Section S‐4‐2, Supporting Information) are investigated. As displayed in Figure 7c, the sandwich‐type cell based on the flexible cathode with VN*_x_*O*_y_* bowl‐like complex achieves good cycling stability at a series of current densities from 1 to 100 A g^−1^. The excellent cycling property and high capacity retention demonstrate its superior high rate capability and cycling stability. In addition, the energy/power densities of the cells are investigated. As displayed in the Figure 7b, the cells based on different structured biocomposites achieve superior volumetric energy and power densities than the other electrochemical devices, such as the Zn//Ni@textile,^33^ Zn//MnO_2_@textile,^33^ Zn//Zn_2_V_2_O_7_@C and Zn//V_2_O_5_@C fiber,^34^ Zn/Ni yard,^35^ ZnO//NiO@carbon cloth,^36^ NiO//Fe_3_O_4_,^37^ Li thin‐film battery,^38^ and so on. The superior high volumetric power densities of the prepared cells are even comparable to commercial supercapacitors, such as TiO_2_//MnO_2_ 39 and AC//AC.^40^ Accordingly, the above results demonstrate the superior energy storage and high power capabilities of both devices. As discussed above, it can be attributed to the unique biodirected structures and superior properties of the vanadate oxynitride nanoparticles. More careful investigations reveal the different features for different cells. As the power density is lower than 0.1 mW cm^−3^, the cell based on the BIS sample achieves the highest energy density among the samples, indicating its high energy storage property in the low power density range. On the other hand, as the power density is higher than 0.1 mW cm^−3^, the cell based on the bowl‐like sample exhibits the highest energy densities among the samples, which demonstrates its superior high rate capability and fast charge/discharge capability. The different features are associated with the different structures of the biocomposites. The compact structure of BIS sample facilitates to high energy storage; while, the thin shell and big voids in the bowl‐like sample enable the fast kinetics and superior high rate capability. Moreover, the thin‐film microbatteries are fabricated based on the interdigitated microelectrodes (Section S‐4‐3, Supporting Information). The enhanced operating potentials of three connected single cells are displayed in Figure 7d. It certifies the possibility of integrating the flexible cells to meet the requirements of the flexible electronics with extended energy output. In addition, the good cycling stability of the microcell under different outside deformations, such as the flat, bending and curving, further demonstrates its good flexibility, high reliability, and excellent cycling stability (Figure 7e).

To demonstrate the potentials of the applications, both kinds of the cells, i.e., the sandwich battery and the microtype battery, are employed to supply the wearable (Figure 7g–i) and implantable (Figure 7f–iii) electronics. The sandwich battery with three cells connected in series is attached the smart glass model, which can well supply the LED light (Figure 7gii). In addition, the cells attached to a pair of trousers can well supply the electronic clock under different degrees of curving with going down on the knee (Figure 7giii–vi). Both results demonstrate the good pliability and high reliability of the sandwich battery for wear able electronic devices. Moreover, the microtype batteries are prepared (Section S‐4‐3, Supporting Information) and implanted to the dorsal of the living Sprague Dawley (SD) rats (Section S‐4‐5, Supporting Information) to reveal its potential in the field of medicine. The implantable battery can well power the LED lights as it is just implanted (Figure 7fiii) and after wound is healed (Figure 7fiv). Accordingly, the results demonstrate the good in vivo performance of microbattery, which enables it to be a good energy storage devices for long‐term in vivo diagnose.

## 3 Conclusion

In this work, we reported a novel biochemistry‐directed strategy based on the controlled reproduction mode to construct intricate hierarchical structures for functional materials. For the first time, the sexual or asexual mode of the bio‐organisms is controlled to build diverse biodirected structures. As a case study, the *S. cerevisiae* and vanadate oxynitrides are employed as biotemplate and active materials. Through controlling the reproduction modes, two different structures of bowl‐like hollow hemisphere and BIS hierarchical structure are constructed for the VN*_x_*O*_y_*/C biocomposites. For both biocomposites, the VN*_x_*O*_y_* nanoparticles are encapsulated in the highly conductive and porous biomass derived carbon networks. It results in their common advantages on the fast kinetics, excellent charge/discharge properties and stable cycling property. Moreover, the different features of diverse biodirected structures result in the different physicochemical characteristics. On the one hand, the bowl‐like sample with thin shell and big void between folded double layers achieves the high surface area, which facilitates the superior high‐rate and high‐power property; On the other hand, the BIS sample with hierarchical and compacter structure is favorable to achieving high energy storage capability. Moreover, the flexible Zn‐ion batteries are fabricated based on the biocomposites. The advantages such as the superior energy/power properties, good pliability, and high reliability ensure their application in multiple fields, such as the wearable electronics, biocompatible electronics, and in vivo long‐term diagnose.

## 4 Experimental Section

The materials, methods, and experimental details are available in the Supporting Information. All the experiments performed with the volunteers were conducted with informed consent from the volunteers. All the implantation operations were performed with the permission of Heilongjiang Administration of Laboratory Animals and strictly in accordance to the nation standard Laboratory Animal Requirements of Environment and Housing Facilities (GB 14925‐2001).

## Conflict of Interest

The authors declare no conflict of interest.

## Supporting information

Supporting Information
