# Supplementary material for: Unlocking the Door of Boosting Biodirected Structures for High‐Performance VNxOy/C by Controlling the Reproduction Mode
Source: Adv Sci (Weinh). 2020 Jan 21;7(5):1903276. doi: 10.1002/advs.201903276 (PMC7055558; doi:10.1002/advs.201903276)
Supplement: Supplementary file 1 — Supporting Information [file ADVS-7-1903276-s001.pdf]

Copyright WILEY-VCH Verlag GmbH & Co. KGaA, 69469 Weinheim, Germany, 2020.

ADVANCED SCIENCE
Open Access

Supporting Information

for *Adv. Sci.,* DOI: 10.1002/advs.201903276

Unlocking the Door of Boosting Biodirected Structures for High-Performance VN_xO_y/C by Controlling the Reproduction Mode

Ting Li, Jing Wang, Xia Li, Liang Si, Sen Zhang,* and Chao Deng**

Copyright WILEY-VCH Verlag GmbH & Co. KGaA, 69469 Weinheim, Germany, 2019.

Supporting Information

Unlocking the Door of Boosting Bio-Directed Structures for High-Performance VN_xO_y/C by Controlling the Reproduction Mode

Ting Li, Jing Wang, Xia Li, Liang Si, Sen Zhang*, and Chao Deng**

Part I: Experimental details, Calculations & Discussions

S-1. Synthesis

S-1-1. Yeast cultivation

S. cerevisiae cells were obtained from the Harbin Academy of Agricultural Sciences and they were cultivated in the Bio-organism Artificial Cultivation Laboratory of Harbin Normal University (HANU, 126°42'E, 45°24'N). The cultivations were carried out on two kinds of biological mediums, *i. e.* the YPD type and the McClary type medium, to grow in different reproduction processes. The YPD type medium is composed of 2 wt.% of Tryptone (Oxoid, England), 1 wt.% of yeast extract (Macklin, China), 2 wt.% of glucose (Macklin, China). The McClary type medium is composed of 0.25 wt.% of yeast extract (Macklin, China), 0.1 wt.% of glucose (Macklin, China), 0.18 wt.% of potassium chloride (Aladdin, China), 0.82 wt.% of sodium acetate (Aladdin, China). The activated cells were inoculated into YPD and McClary mediums, respectively. Then they were incubated on a rotary shaker to grow to an optical density. After washing and centrifuged in distilled water to remove the unwanted impurities from the harvest cells, the purified and concentrated cells were collected for further use.

S-1-2. Preparation of bio-precursors

Firstly, the precursor solution is prepared. The V_2O_5 power (Mackin, China) is dissolved in the mixed solution of H_2O_2 and distilled water under vigorous stirring. The obtained transport solution is

used as the precursor solution. Next, the bio-precursors are prepared. The different kinds of yeast cells from asexual and sexual reproduction modes are added into the prepared precursor solution under magnetic stirring. The suspensions are firstly maintained at room temperature and then at elevated temperature of 40 ^{o}C. After that, the suspensions are centrifuged, frozen completely in liquid nitrogen and freeze-dried. The resultant powders are denoted as the bio-precursors.

S-1-3. Preparation of bio-composites

Firstly, both kinds of the bio-precursors are annealed at 700 ^{o}C in nitrogen atmosphere to carbonize the bio-organism bases. Then the intermediate products are recalcinated at 400 ^{o}C in the air to achieve the intermediate products. Finally, the intermediate products are re-annealed in NH_3 atmosphere to achieve the final product.

S-1-4. Preparation of the reference samples

i) The precursor solution in S-1-2 was dried at 80 ^{o}C in an oven overnight. The obtained powder is annealed at 400 ^{o}C in air to achieve the V_2O_5 RF sample. Then the V_2O_5 RF sample is annealed in NH_3 atmosphere to achieve the VN_xO_y RF sample.

ii) The RF/AC reference sample was prepared by mixing the RF reference sample with the activated carbon in the agate mortar to achieve the same carbon content as the bio-composites.

S-1-5. Synthesis of the bio-composite/SWNT flexible cathodes

Single wall carbon nanotubes (SWNT) were purchased from Nanjing XFNANO Materials Tech Co., Ltd. The powder of the bio-composites, the single-walled carbon nanotubes and the PVDF binder were mixed in the NMP solution, which was then put into ultrasonicating bath for one hour. The resultant uniform ink was casting onto the surface of a piece of glass, which was then dried at room temperature to achieve the bio-composite/SWNT flexible cathodes.

S-2. Materials characterizations

Powder X-ray diffraction (XRD, Bruker D8/Germany) using Cu Kα radiation was employed to identify the crystalline phase of the material. The morphology was observed with a scanning electron microscope (SEM, HITACHIS-4700) and a transmission electron microscope (TEM, JEOS-2010 PHILIPS). The element distribution of the sample was confirmed by energy dispersive X-ray detector (EDX). XPS analyses (XPS, Thermo escalab 250 Xi) were conducted employing Al Kα radiation. Nitrogen adsorption-desorption isotherms were measured using a Micromeritics ASAP 2010. Sample preparation included degassing at 378 K (105 ^{o}C) for 10 h in a vacuum of 10^{-6} Torr. Inductively coupled plasma spectrometry (ICP, Agilent 7500CX) was employed to analyze the element content in the samples.

S-3. Electrochemical measurements

The electrodes are made by mixing the active material, the carbon black and the binder in a weight ratio of 7:2:1. The mixture was casting on the titanium foil and vacuum drying at 80 ^{o}C overnight to achieve the working electrode. Zn foil was used as the counter electrode and the $Zn(CF_3SO_3)_2$ aqueous solution was used as the electrolyte. The cyclic voltammetry (CV) were measured in a CHI electrochemical workstation, and the galvanostatic charge/discharge tests were conducted on a LAND battery testing system (Wuhan, China).

S-4. Fabrication and characterization of the flexible solid-state-battery

S-4-1. Preparation of the Zn nanosheet/SWNT anode

Firstly, the single-wall carbon nanotube (SWNT) based film was prepared. The SWNT-ink was prepared by dispersing the single-walled carbon nanotubes and the PVDF in the NMP solution, and then it is put into ultrasonicating bath for one hour. The resultant uniform ink was casting onto the surface of a piece of glass, which was then dried at room temperature. After it is separated from the substrate, the flexible SWNT film was obtained. Next, Zn nanosheets were grown on the SWNT film by electrodeposition at room temperature employing the CHI 760 electrochemical work station. The

electrodeposition was carried out in a three-electrode configuration with the textile as working electrode, a Pt plate as counter electrode and a saturated calomel electrode (SCE) as reference electrode. The mixed solution of $ZnSO_4 \cdot 7H_2O$, Na_2SO_4 and H_3BO_3 was used as electrolyte. The electrodeposition was conduct at -40 mA cm^{-2} at room temperature. Then the resultant electrode was washed with DI water and dried in a vacuum oven at 60 ^{o}C for 12 hours.

S-4-2. Fabrication of the sandwich-type flexible Zn-ion battery

The sandwich-type battery was assembled by the bio-composite/SWNT cathode, Zn nanosheet/SWNT anode and the polymer gel electrolyte. Firstly, the commercial gelatine and potassium persulfate were dissolved in the mixed solution under stirring at 80 ^{o}C. Then the acryamide and N, N′-methylenebisacrylamide were added to the mixture for another three hours stirring. Afterwards, the mixture was injected into the flexible electrodes and assembled the sandwich type cell. Then the cell was soaked again in the mixture solution for another two hours to achieve the equilibrated state.

S-4-3. Fabrication of the micro-type Zn-ion battery

i) Synthesis of interdigitated microelectrodes. The films were laser-ascribed using a commercial laser marking machine with the maximum power of 5 W. The digital computerized control system is used to control the structure of the electrode. The interdigitated structure is built to prevent short circuit between two adjacent microelectrodes.

ii) Fabrication of thin-film micro-battery. The thin-film microbattery was fabricated based on the interdigitated electrodes. The microelectrodes were soaked in the aqueous electrolyte for one day, and then they were encapsulated by the polymer gel electrolyte as described above. After that, the microbattery was soaked again in the aqueous electrolyte for another two hours to achieve the equilibrated state.

iii) Encapsulated by SF hydrogel. The microbattery battery was encapsulated by the SF hydrogel to get good biocompatibility for implanting. The silk fibroin (SF) solution was prepared using cocoons following a standard protocol. Firstly, the silkworm cocoon was cut into pieces and boiled in aqueous solution of Na_2CO_3 for half an hour for degumming. Then the silk fibers were washed and solubilized in lithium bromide (LiBr) solution and the resultant solution was dialyzed against deionized water for removal of traces of LiBr followed by centrifugation to yield silk fibroin solution. Next, the silk fibroin/PVP (polyvinyl pyrrolidone) IPN (interpenetrating polymer network) hydrogel was prepared. The resultant solution was mixed with the prepared SF solution and desirable salt under stirring. Then the microbattery was immersed into the solution to achieve the surface coating.

S-4-4. Electrochemical measurements of the flexible battery

The flexible batteries are prepared as described in S-4-2 and S-4-3. Then the charge/discharge tests were carried on the batteries by Land battery system.

S-4-5. *In vivo* implantation of microbattery

All the implantation operations were performed strictly in accordance to "Heilongjiang Administration Rule of Laboratory Animals" and the nation standard "Laboratory Animal Requirements of Environment and Housing Facilities (GB 14925-2001)." Before the implantation operation, the microbattery and all the surgical instruments were sterilized by Gamma-ray irradiation and autoclaving methods. The SD rats were anaesthetized with the intraperitoneal injection of pentobarbital sodium. The as-sterilized microbatteries were implanted into the subcutaneous region of the SD rats. The wound was instantly sutured after implantation.

S-5. Calculation of the electrochemical parameters

(i) Specific capacity (Q)

The specific capacity is calculated based on the galvanostatic charge/discharge curves,

$$Q = \frac{I \times \Delta t}{m} \qquad (1)$$

Where Q is the specific capacity, Δt is the discharge time, I is the discharge current, m is the mass of the active material in the single electrode.

(ii) Relative capacity during initial a few cycles (Q_c)

The relative capacity during cycling is calculated based on the following equation,

$$Q_c = \frac{Q_i}{Q_{60}} \qquad (2)$$

Where Q_c is the relative capacity during initial a few cycles, Q_i is the capacity at a certain cycle, Q_{60} is the capacity at the 60th cycle.

(iii) Relative rate capacity (Q_r)

The relative capacity of the single electrode is calculated based on the following equation,

$$Q_r = \frac{Q_i}{Q_{10}} \qquad (3)$$

Where Q_r is the relative rate capacity, Q_i is the capacity at a certain current density, Q_{10} is the capacity at 10 A g^{-1}.

(iv) Cycling retention (C_r)

The cycling retention is calculated based on the following equation,

$$C_r = \frac{Q_a}{Q_b} \times 100\% \qquad (4)$$

Where C_r is the capacity retentions after cycling, Q_a is the capacity of the electrode in the last cycle, Q_b is the capacity of the electrode in the initial cycle.

(v) Normalized areal capacity (Q_n)

The normalized areal capacity of the single electrode is calculated based on the following equation,

$$Q_n = \frac{Q_i}{Q_l} \qquad (5)$$

Where Q_n is the normalized areal capacity, Q_i is the areal capacity at a certain current density, Q_l is the areal capacity at the lowest current density.

S-6. Calculation of the capacitive contribution

The calculation of the capacitive contributions is based on the CV curves measured at a series of scan rate. In the CV curves, the current is believed to be originated from two independent and distinct parts: the surface-induced capacitive process and the diffusion-controlled process. As described in the follow equation,

$$i = a \times v^b \qquad (6)$$

Where *i* is the measured current; *v* is the scan rate; both *a* and *b* are adjustable parameters. Determined from the slope of the curve between the log*i* versus log*v*, the *b* values of different peaks can be achieved.

$$\log i = \log a + b \log v \qquad (7)$$

The *b* values change from 0.5 to 1. When *b* value is 0.5, it indicates a diffusion-controlled process; as *b* value is 1.0, it suggests a complete capacitive process. To further characterize each contribution to the total properties, the measured current (*i*) at a fixed potential can be separated into two functions of the capacitive and the diffusion processes as described in the following,

$$i = k_1 v + k_2 v^{1/2} \qquad (8)$$

Where the k_1v and $k_2v^{1/2}$ correspond to the capacitive and diffusion contributions, respectively. Based on the above equation, the capacitive contribution ratios at various scan rates can be quantitatively achieved.

Part II: Supporting Figures

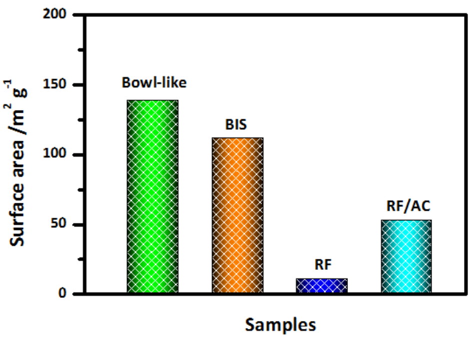

Figure S1. Comparison of the surface areas of the V_2O_5 based samples. The RF/AC sample is prepared by mixing the RF sample with the activated carbon to achieve the same carbon content as the bio-composites.

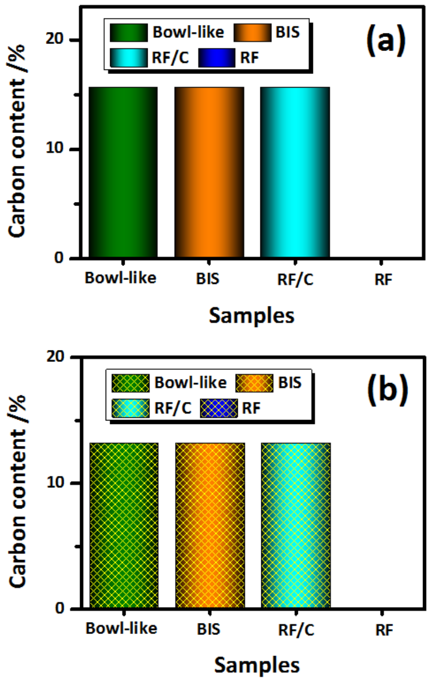

Figure S2. Carbon contents of the vanadate oxide based (a) and vanadate oxynitride (b) based samples with bowl-like and BIS structures, RF reference samples and the RF/C samples. For both kinds of vanadate complex, the bowl-like, BIS bio-directed samples and the RF/C reference samples have the same carbon contents. Whilst, there is no carbon detected in the RF reference samples. The RF/C reference samples are prepared by mixing the RF reference samples with the activated carbon to achieve the same carbon content as the bio-directed samples.

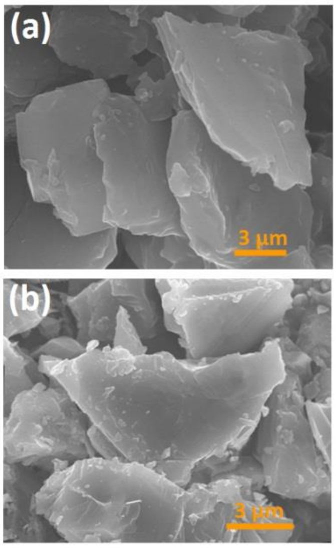

Figure S3. SEM images of vanadate oxide based (a) and vanadate oxynitride (b) based RF reference samples.

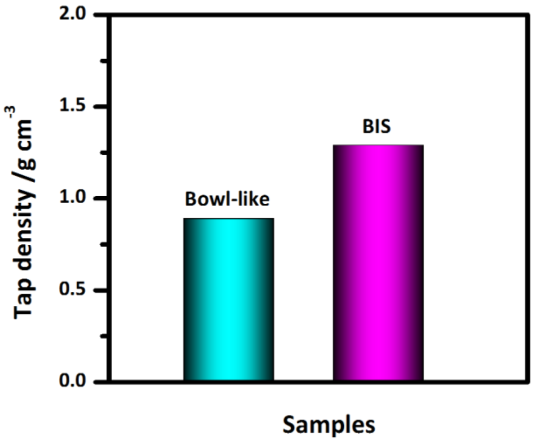

Figure S4. Comparisons of the tap densities of the bowl-like and BIS structured carbon substrates. Both kinds of the carbon substrates are prepared by dissolving the active materials from the prepared bio-composites.

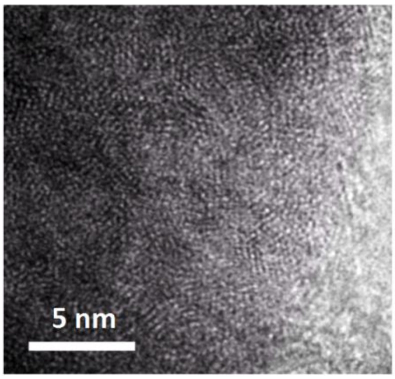

Figure S5. HRTEM image of the fully charged product after initial charging process. The image reveals the existence of abundant defects and the absence of well-resolved lattice fringe that has been observed for the pristine sample (insert of Figure 2p). The result demonstrates the formation of defect-rich product after the initial charging process.

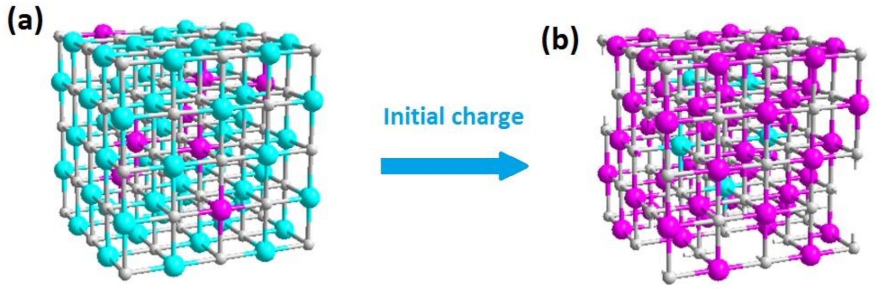

Figure S6. Schematic illustration of the pristine VN_xO_y ($VN_x(O_{poor})_y$) with close-packed face-centered cubic structure that limits Zn ion diffusion (a) and the new product ($VN_x(O_{rich})_y$) after initial charging (b) with the disordered structure and abundant defects. It can be seen that the vanadate oxynitride undergoes a conversion reaction during the initial charge, where high valent anion nitrogen were partially replaced by low valent oxygen. The conversion reaction produce the new product (b) with anion disordered structure and abundant defects, which facilitates the Zn ion diffusion. The blue, purple and white spheres represent the nitrogen, oxygen and vanadate atom, respectively.

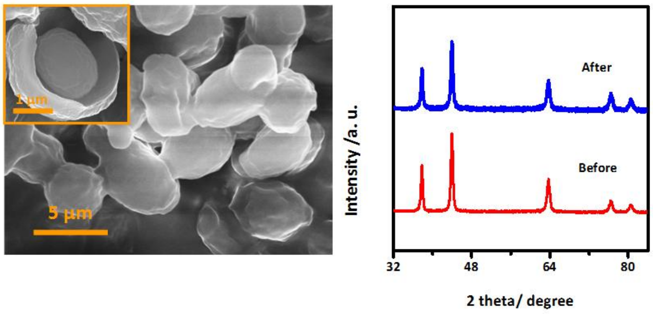

Figure S7. SEM image (a) and XRD patterns (b) of VN_xO_y based BIS sample after long-term cycling. In a, a partially broken particle is showed as insert. In b, the patterns between the samples before and after cycling are compared. Both the well-retained morphology and phase structure of the cycled sample contribute to the superior long-term cycling properties of the VN_xO_y based sample.

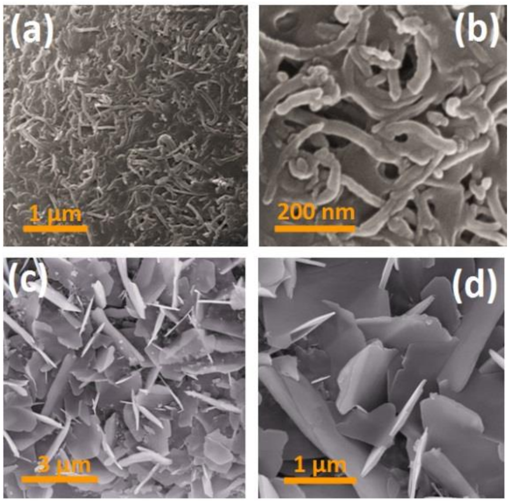

Figure S8. SEM image of the Zn nanosheet/SWNT flexible electrode. (a, b) the SWNT substrate; (c, d) the Zn nanosheet on the substrate.
